# Dual Trigger for Final Follicular Maturation Improves Cumulative Live-Birth Rate in Ovarian Stimulation for Freeze-All *In Vitro* Fertilization/Intracytoplasmic Sperm Injection Cycles

**DOI:** 10.3389/fendo.2021.708247

**Published:** 2021-07-19

**Authors:** Haiyan Zhu, Chenqiong Zhao, Yibin Pan, Hanjing Zhou, Xiaoying Jin, Wen Xu, Songying Zhang

**Affiliations:** ^1^ Assisted Reproduction Unit, Department of Obstetrics and Gynaecology, Sir Run Run Shaw Hospital, School of Medicine, Zhejiang University, Hangzhou, China; ^2^ Department of Obstetrics and Gynecology, Key Laboratory of Reproductive Dysfunction Management of Zhejiang Province, Hangzhou, China

**Keywords:** dual trigger, freeze-all, cumulative live birth, hCG, *in vitro* fertilization

## Abstract

**Study Question:**

Does dual trigger in freeze-all *in vitro* fertilization (IVF)/intracytoplasmic sperm injection (ICSI) cycles improve the cumulative live-birth outcome compared with human chorionic gonadotropin (hCG) trigger?

**Summary Answer:**

Dual trigger for final follicular maturation improves the cumulative pregnancy and live-birth rates compared with hCG trigger in freeze-all IVF/ICSI cycles.

**What Is Known Already:**

Dual trigger could increase the numbers of oocytes and mature oocytes and improve pregnancy rates.

**Study Design, Size, Duration:**

This retrospective cohort analysis included data from 4438 freeze-all IVF/ICSI cycles between January 2012 and December 2017.

**Participants/Materials, Setting, Methods:**

Women aged 20−49 years who underwent ovarian stimulation and oocyte retrieval for autologous IVF/ICSI with a freeze-all policy in our centre were enrolled. Data on number of oocytes retrieved, number of mature oocytes, clinical pregnancy rate, live-birth rate, cumulative pregnancy rate, and cumulative live-birth rate (CLBR) were assessed and compared between patients who underwent a dual trigger and hCG trigger. Multivariate logistic regression was performed to identify and adjust for factors known to independently affect the CLBR.

**Main Results and the Role of Chance:**

A total of 4438 IVF/ICSI cycles were analyzed, including 1445 cycles with single hCG trigger and 2993 cycles with dual trigger. The cumulative biochemical pregnancy rate (60.8% *vs.* 68.1%, P<0.001; odds ratio (OR): 0.727; 95% confidence interval (CI): 0.638–0.828), cumulative clinical pregnancy rate (52.9% *vs.* 58.5%, P<0.001; OR: 0.796; 95%CI: 0.701–0.903), and CLBR (44.3% *vs.* 50.5%, P<0.001; OR: 0.781; 95%CI: 0.688–10.886) were all significantly lower in the hCG-trigger group compared with the dual-trigger group. The clinical pregnancy rate (48.2% *vs.* 58.2%, P=0.002; OR: 0.829; 95%CI: 0.737–0.934) and embryo implantation rate (34.4% *vs.* 38.9%, P<0.001; OR: 0.823; 95%CI: 0.750–0.903) in each transfer cycle were also significantly lower in the hCG-trigger group compared with the dual-trigger group. After controlling for all potential confounding variables, the trigger method was identified as an independent factor affecting the CLBR. The OR and 95%CI for hCG trigger were 0.780 and 0.641–0.949 (P=0.013).

**Limitations, Reasons for Caution:**

The data used to analyse the effect of dual trigger on cumulative pregnancy and live-birth outcomes were retrospective, and the results may thus have been subject to inherent biases. Further prospective randomized controlled trials are required to verify the beneficial effects of dual trigger.

**Wider Implications of the Findings:**

Dual trigger had a positive effect on CLBRs, suggesting that it could be used as a routine trigger method in freeze-all cycles.

**Study Funding/Competing Interest(s):**

This study was supported by grants from National Key Research and Development Program of China (2018YFC1004800), the Natural Science Program of Zhejiang (LY19H040009), the National Natural Science Foundation of China (No. 81601236). No authors have competing interests to declare.

## Highlights

Dual trigger for final follicular maturation improves the cumulative pregnancy and live-birth rates compared with hCG trigger in freeze-all IVF/ICSI cycles.

## Introduction

The rampant spread of COVID-19 worldwide has led to changes in people’s work and life habits. International experts in assisted reproductive technology (ART) have appealed for a “safety first, assisted reproductive therapy second” approach. To avoid frequent contacts with people in hospital and to ensure safety, the flexibilities of embryo transfer and pregnancy schedule have become important considerations for ART in terms of treatment flow and treatment mode. Increasing numbers of ART centres have thus tended to adopt a freeze-all strategy for patients undergoing conventional controlled ovarian hyperstimulation cycles. However, there is currently no consensus regarding the optimal trigger method for freeze-all cycles.

During natural ovulatory cycles, peak oestrogen (>200 pg/ml) secreted by preovulatory follicles induces the release of gonadotropin-releasing hormone (GnRH) from the hypothalamus, resulting in the release of gonadotropin by the pituitary gland and a luteinizing hormone (LH)/follicle-stimulating hormone (FSH) surge. The LH surge induces the resumption of oocyte meiosis and luteinization of granulosa cells, which generates a small amount of progesterone (1). LH/FSH and progesterone synergistically activate proteolytic enzymes in the follicular fluid to digest the collagen layer of the follicle wall and promote ovulation (1). In natural cycles, the LH surge lasts for 48 hours and the plateau is maintained for 14 hours. In contrast, in conventional controlled ovarian hyperstimulation cycles, hCG has traditionally been used to induce final oocyte maturation and has been considered as the gold standard for triggering the final follicular maturation (2). hCG, as a substitute for the natural endogenous LH surge, can induce luteinization of granulosa cells and final oocyte maturation. However, the half-life of hCG is longer than that of endogenous LH and the surge could thus last for 48 hours, while the biological effect could be maintained for several days (1, 3). A single bolus of hCG thus leads to supraphysiological luteal steroid levels and the formation of multiple corpora lutea, resulting in an increased risk of ovarian hyperstimulation syndrome (OHSS) (1, 3). Moreover, hCG administration promotes the release of various vascular factors, such as endothelial growth factor, which increase vascular permeability and aggravate OHSS (4, 5).

Various strategies have been developed to reduce the occurrence and development of OHSS, of which the most effective involves using a GnRH agonist (GnRHa) to trigger final follicle maturation. The GnRHa binds to the GnRH receptor inducing a physiological surge similar to that of LH and FSH, which stimulates the oocyte to dissociate from the follicle wall and undergo nuclear maturation and cumulus expansion. Compared with the natural cycle, the LH surge induced by GnRHa is shorter (about 24–36 hours) and there is no 14-hour plateau (6), causing early luteolysis and corpus luteum dysfunction following GnRHa trigger, which is associated with lower pregnancy and higher abortion rates (7). Intensive luteal support or adjuvant small doses of hCG administered simultaneously with the GnRHa trigger have been suggested to redress luteal phase insufficiency and improve pregnancy rates. The concept of a “dual trigger” that combines one bolus of GnRHa with a standard or reduced dosage of hCG at the time of triggering has accordingly been shown to improve the rates of oocyte recovery, oocyte maturation, pregnancy, and live birth (8, 9). However, the effect of such a dual trigger on clinical outcomes in freeze-all cycles, especially in terms of cumulative pregnancy and live-birth rates, is still unclear.

The present study accordingly investigated the role of the dual-trigger strategy in improving the cumulative pregnancy and live-birth rates in patients undergoing freeze-all cycles.

## Materials and Methods

### Study Design

This was a retrospective analysis including data for 4438 fresh IVF/ICSI cycles at the Centre for Reproductive Medicine of the Sir Run Run Shaw Hospital of Zhejiang University from January 2012 to December 2017. Women aged 20–49 years who underwent ovarian stimulation and oocyte retrieval for autologous IVF/ICSI with a freeze-all policy and underwent frozen embryo transfer in our centre were considered eligible for enrolment. Women who did not deliver a baby but did not use all their embryos were excluded. Only women who either delivered a live infant or who had no remaining frozen embryos after a single stimulation cycle were included in the analysis.

### Ovarian Stimulation Protocol

Recombinant and/or urinary gonadotrophins (rFSH: Gonal-F, 150-225IU, Serono Laboratories, Switzerland/human menopausal gonadotropin: 150-225IU, Anhui Fenyuan Pharmaceutical Co., China) were used for ovarian stimulation. The ovarian stimulation protocol and initiating gonadotrophin dose were determined according to maternal age, number of basal follicles, basic ovarian endocrine test, and body mass index. Utrogestan (100 mg/day, Laboratories Besins International, Paris, France) was added to progestin-primed ovarian-stimulation protocols to inhibit the LH surge during ovarian simulation. Transvaginal ultrasound was used to monitor follicular development. When at least three follicles reached 18 mm in diameter, hCG (5000, 8000, 10000IU, Lizhu Pharmaceutical Trading Co., China) (hCG trigger) or a combination of triptorelin (0.1–0.2 mg, Decapeptyl, Ferring Pharmaceuticals, Germany) and hCG (2000, 5000, 6000IU)(dual trigger) were applied to trigger final oocyte maturation. Oocyte retrieval was conducted under transvaginal ultrasound guidance 36 hours after triggering.

### Frozen Embryo Transfer (FET)

Survival of thawed cleavage embryos was evaluated morphologically immediately after thawing. Embryos with more than half of their cells intact were judged as survived and could be transferred. Three main types of protocols were used to prepare the endometrium, as reported previously (10): the natural cycle, hormone replacement cycle, or human menopausal gonadotropin-stimulated cycle. The preparation type was determined by the patient’s menstrual cycle pattern. Daily intramuscular injection of 60 mg of progesterone plus 20 mg oral progesterone (Duphaston, Abbott, Nertherlands) was started 3 days before day-3-embryo transfer or 5 days before blastocyst transfer, for luteal-phase support. Serum β-hCG was measured 12 days after embryo transfer, and a value >50 IU/ml was considered as a positive pregnancy. Daily intramuscular injections of progesterone were reduced to 40 mg combined with 20 mg oral progesterone, and continued until the 10th week of pregnancy.

### Main Outcome Measures

The primary outcome measure was the short term cumulative live-birth rate (CLBR) per ovarian stimulation cycle. Short term CLBR was defined as delivery in subsequent frozen-thawed cycles, and only the first delivery was included in the analysis (11). Secondary outcome measures were clinical pregnancy rate, implantation rate, and cumulative pregnancy rate.

### Statistical Methods

Continuous variables were presented as the mean ± standard deviation and were compared using *t*-tests. Categorical variables were presented as number of cases, corresponding percentages, and odds ratios (ORs) and 95% confidence intervals (CIs), and were analysed using χ^2^ and Fisher’s exact tests.

Binary logistic regression was performed for multivariate analysis of the following potential predictors: trigger method, maternal age, number of oocytes retrieved, duration of stimulation, number of good quality embryos, and insemination method. The regression model results were presented as OR and 95%CIs. All analyses were performed using SPSS 22.0 (SPSS Inc., Chicago IL, USA).

## Results

### Patient Characteristics

A total of 4438 IVF/ICSI cycles were analysed, including 1445 single-hCG trigger and 2993 dual-trigger cycles. The baseline characteristics of the patients are presented in **Table 1**. There were no significant differences in maternal age and aetiology of infertility between the two groups. Compared with the dual-trigger group, the hCG-trigger group had higher basal FSH (8.6 ± 3.5 *vs*. 8.0 ± 3.2, P<0.001) and higher basal E2 (47.6 ± 29.3 *vs*. 42.7 ± 27.7, P<0.001) levels, but there was no significant difference in basal LH levels (4.8 ± 4.2 *vs*. 4.9 ± 5.8, P=0.38). In addition, the duration of infertility was significantly longer in the hCG-trigger compared with the dual-trigger group (4.2 ± 3.4 *vs*. 3.7 ± 3.0, P<0.001). The hCG-trigger group also underwent more micro-stimulation protocols and fewer progestin-primed ovarian stimulation protocols, and the duration of stimulation was accordingly significantly shorter in the hCG-trigger than in the dual-trigger group (7.5 ± 2.9 *vs*. 9.1 ± 2.3, P<0.001). Furthermore, the hCG dosage was significantly higher in the hCG-trigger group compared with the dual-trigger group (8520.6 ± 2063.0 *vs*. 49204.7 ± 609.1, P<0.001).

**Table 1:** Basal characteristics and fresh IVF/ICSI outcomes.

	hCG trigger	Dual trigger	Statistic	P value	OR (95% CI)
No. of stimulation cycles, n (%)	1445 (32.6%)	2993 (67.4%)			
Age (mean ± SD)	33.7 ± 5.4	33.3 ± 4.9	1.87	0.062	
D3 FSH (IU/L) (mean ± SD)	8.6 ± 3.5	8.0 ± 3.2	5.179	<0.001	
D3 E2 (ng/L) (mean ± SD)	47.6 ± 29.3	42.7 ± 27.7	-5.124	<0.001	
D3 LH (IU/L) (mean ± SD)	4.8 ± 4.2	4.9 ± 5.8	-0.878	0.38	
Infertility factor, n (%)			38.116	<0.001	
Tubal factor	938 (64.9%)	1867 (62.4%)	2.692	0.104	1.116 (0.979-1.272)
Endometriosis	76 (5.3%)	118 (3.9%)			
Ovulatory disorder	72 (5.0%)	87 (2.9%)			
Diminished ovarian reserve	124 (8.6%)	405 (13.5%)			
Male factor	174 (12.0%)	364 (12.2%)			
Unexplained	61 (4.2%)	152 (5.1%)			
Duration of infertility (years)	4.2 ± 3.4	3.7 ± 3.0	4.834	<0.001	
Stimulation protocol, n (%)			833.041	<0.001	
Micro-stimulation protocols	821 (56.8%)	873 (29.2%)			
Progestin-primed ovarian stimulation protocols	200 (13.8%)	1780 (59.5%)			
GnRH antagonist protocols	424 (29.3%)	340 (11.4%)			
Duration of stimulation (day)	7.5 ± 2.9	9.1 ± 2.3	-19.433	<0.001	
Dose of hCG trigger	8520.6 ± 2063.0	4904.7 ± 609.1	88.062	<0.001	
No. of oocytes retrieved	6.0 ± 5.6	6.4 ± 5.2	-2.335	0.023	
Oocytes recovery rate, n (%)	8663/10988 (78.8%)	19152/24271 (78.9%)	0.021	0.888	0.996 (0.942-1.052)
MII rate, n (%)	3015/3324 (90.7%)	6016/6589 (91.3%)	0.98	0.331	0.929 (0.804-1.074)
Use of ICSI, n (%)	642 (44.4%)	1062 (35.5%)	32.974	<0.001	1.454 (1.279-1.652)
Fertilization rate (%)					
IVF	3819/5339 (71.5%)	8865/12563 (70.6%)	1.693	0.196	1.048 (0.976-1.125)
ICSI	2431/3015 (80.6%)	4658/6016 (77.4%)	12.209	<0.001	1.214 (1.089-1.353)
Good quality embryo rate (%)	3086/6165 (50.1%)	6345/13286 (47.8%)	8.917	0.003	1.096 (1.032-1.165)

### Fresh Cycle Outcomes

The ovarian stimulation and IVF/ICSI outcomes are shown in **Table 1**. The number of oocytes was significantly lower in the hCG-trigger group compared with the dual-trigger group (6.0 ± 5.6 *vs*. 6.4 ± 5.2, P=0.023), but there was no significant difference in oocyte recovery rate (78.8% *vs*. 78.9%, P=0.888; OR: 0.996; 95%CI: 0.942–1.052). Furthermore, although the oocyte maturation rate was lower in the hCG-trigger compared with the dual-trigger group, the difference was not significant (90.7% *vs*. 91.3%, P=0.331; OR: 0.929; 95%CI: 0.804–1.074). The proportion of cycles of ICSI was significantly higher in the hCG-trigger group (44.4% *vs*. 35.5%, P<0.001; OR: 1.454; 95%CI: 1.279–1.652). There was no significant difference in IVF fertilization rates between the two groups, but the ICSI fertilization rate was significantly higher in the hCG-trigger compared with the dual-trigger group (80.6% *vs*. 77.4%, P<0.001; OR: 1.214; 95%CI: 1.089–1.353). The rate of good-quality embryos was also significantly higher in the hCG-trigger group (50.1% *vs*. 47.8%, P=0.003; OR: 1.096; 95%CI: 1.032–1.165).

### Frozen Cycle Outcomes

To analyse the impact of the dual-trigger approach on the clinical outcome of frozen-thawed cycles, we compared the outcome of FET after dual trigger with that after hCG trigger (**Table 2**). Day-3-embryo transfer was performed in 92.1% of cycles in the hCG group and 85.9% of cycles in the dual-trigger group (P<0.001, OR: 1.905, 95%CI: 1.553–2.336), but the mean number of transferred embryos did not differ significantly between the two groups. The transfer rate of good-quality embryos was significantly higher in the hCG-trigger group compared with the dual-trigger group (66.5% *vs*. 61.7%, P<0.001; OR: 1.233; 95%CI: 1.123–1.353). However, the clinical pregnancy rate (48.2% *vs*. 58.2%, P=0.002; OR: 0.829; 95%CI: 0.737–0.934), embryo implantation rate (34.4% *vs*. 38.9%, P<0.001; OR: 0.823; 95%CI: 0.7500.903), and live-birth rate (26.6% *vs*. 31.7%, P<0.001; OR: 0.783; 95%CI: 0.7090.864) were all significantly lower in the hCG-trigger compared with the dual-trigger group. Regarding neonatal outcomes, the preterm birth rate was significantly lower in the hCG-trigger group compared with the dual-trigger group (11.8% *vs*. 17.8%, P<0.001; OR: 0.619; 95%CI: 0.470–0.815). There were no significant differences in neonatal weight, low-birth-weight ratio, and foetal sex ratio between the two groups.

**Table 2:** Frozen cycle outcomes.

	hCG trigger	Dual trigger	Statistic	P value	OR (95% CI)
No. of stimulation cycles,n (%)	1445 (32.6%)	2993 (67.4%)			
Total number of thawing cycles (n)	1655	3357			
Total number of embryo transfer cycles (n)	1642	3340			
Day-3 embryo transfer, n (%)	1512 (92.1%)	2870 (85.9%)	39.365	<0.001	1.905 (1.553-2.336)
Blastocyst transfer, n (%)	130 (7.9%)	470 (14.1%)			
Mean number of blastocysts transferred, mean (SD)	1.5 ± 0.5	1.5 ± 0.5	-0.102	0.919	
Mean number of Day-3 embryos transferred, mean (SD)	1.8 ± 0.5	1.8 ± 0.5	-0.657	0.511	
No. of good quality embryos transferred, n/n (%)	1941/2918 (66.5%)	3644/5905 (61.7%)	19.431	<0.001	1.233 (1.123-1.353)
Endometrial thickness (mm)	9.2 ± 1.6	9.4 ± 2.8	-3.013	0.003	
Clinical pregnancy rate per transfer cycles, n (%)	791/1642 (48.2%)	1765/3340 (52.8%)	9.615	0.002	0.829 (0.737-0.934)
Implantation rate per transferred embryos (%)	1004/2918 (34.4%)	2299/5905 (38.9%)	17.08	<0.001	0.823 (0.750-0.903)
Live birth rate per transferred embryos, n (%)	777/2918 (26.6%)	1870/5905 (31.7%)	23.625	<0.001	0.783 (0.709-0.864)
Multiple pregnancy rate per delivery, n/n (%)	142/635 (22.4%)	381/1489 (25.6%)	2.571	0.111	0.835 (0.671-1.041)
Male infants (%)	413/777 (53.2%)	920/1870 (49.2%)	3.435	0.066	1.172 (0.991-1.385)
Live birth weight of singleton pregnancies (g)	3260.6 ± 549.6	3287.6 ± 530.1	-0.929	0.353	
Live birth weight of twin pregnancies (g)	2507.5 ± 458.1	2466.6 ± 468.4	0.889	0.374	
Preterm birth <37 weeks, n (%)	75/635 (11.8%)	265/1489 (17.8%)	11.864	<0.001	0.619 (0.470-0.815)
LBW <2,500 g	152/777 (19.6%)	408/1870 (21.8%)	1.675	0.21	0.871 (0.707-1.073)
VLBW <1,500 g	11/777 (1.4%)	28/1870 (1.5%)	0.025	1	0.945 (0.468-1.907)

### Cumulative Pregnancy and Live-Birth Rates

We compared the cumulative pregnancy and live-birth rates between the hCG and dual-trigger groups (**Table 3**). Compared with the dual-trigger group, the cumulative biochemical pregnancy rate (60.8% *vs*. 68.1%, P<0.001; OR: 0.727; 95%CI: 0.638–0.828), cumulative clinical pregnancy rate (52.9% *vs*. 58.5%, P<0.001; OR: 0.796; 95%CI: 0.701– 0.903), and CLBR (44.3% *vs*. 50.5%, P<0.001; OR:0.781; 95%CI: 0.688–10.886) were all significantly lower in the hCG-trigger group. We also analysed the impact of different trigger methods on cumulative pregnancy rates and CLBRs in older women (>35 years). Among patients aged >35 years, the cumulative biochemical pregnancy rate (45.0% *vs*. 52.2%, P=0.01; OR: 0.750; 95%CI: 0.605–0.931) and CLBR (23.3% *vs*. 29.1%, P=0.019; OR: 739; 95%CI: 0.576–0.947) were also significantly lower in the hCG-trigger group.

**Table 3:** Cumulative reproductive outcome of one complete cycle of fresh and frozen–thawed embryo transfer until the first live birth in women.

Cumulative reproductive outcome	hCG trigger	Dual trigger	Statistic	P value	OR (95% CI)
Total					
Positive p-hCG (≥50 IU/L), n (%)	879/1445 (60.8%)	2039/2993 (68.1%)	23.031	<0.001	0.727 (0.638-0.828)
Ongoing pregnancy, n (%)	764/1445 (52.9%)	1751/2993 (58.5%)	12.585	<0.001	0.796 (0.701-0.903)
Live birth, n (%)	640/1445 (44.3%)	1510/2993 (50.5%)	14.808	<0.001	0.781 (0.688-0.886)
> 35 years					
Positive p-hCG (≥50 IU/L), n (%)	236/524 (45.0%)	475/910 (52.2%)	6.819	0.01	0.750 (0.605-0.931)
Ongoing pregnancy, n (%)	181/524 (34.5%)	354/910 (38.9%)	2.701	0.112	0.829 (0.662-1.037)
Live birth, n (%)	122/524 (23.3%)	265/910 (29.1%)	5.753	0.019	0.739 (0.576-0.947)

### Multivariate Logistic Regression Analysis for CLBR

We conducted logistic regression analysis to clarify the influence of dual trigger on the CLBR (**Table 4**). After controlling for all potential confounding variables, trigger method, maternal age, number of oocytes retrieved, and number of good-quality embryos were identified as independent predictive factors affecting the CLBR. The ORs (95%CIs) were 0.780 and 0.641–0.949 (P=0.013) for the hCG trigger, 1.055 (1.028–1.083, P<0.001) for the number of oocytes retrieved, 0.890 (0.872–0.908, P<0.001) for age, 1.364 (1.273–1.462, P<0.001) for the number of good quality embryos, and 1.053 (1.011–1.097, P=0.013) for endometrial thickness. The insemination method, basal FSH and E2 levels, the infertility duration, and the duration of stimulation had no significant effect on the CLBR.

**Table 4:** Logistic regression analysis with odds ratios for the CLBR.

Cumulative live birth	Odds ratio	B	95% confidence interval	P value
Tigger				
hCG trigger	0.780	-0.249	0.641-0.949	0.013
Dual trigger	1			
Age	0.89	-0.117	0.872-0.908	<0.001
Oocytes	1.055	0.054	1.028-1.083	<0.001
No. of good quality embryos	1.364	0.311	1.273-1.462	<0.001
Endometrial thickness	1.053	0.052	1.011-1.097	0.013
Insemination method				
IVF	0.949	-0.052	0.7951-1.133	0.565
ICSI	1			
Basal FSH level	0.998	-0.002	0.967-1.030	0.92
Basal E2 level	0.999	-0.001	0.995-1.002	0.377
Infertility duration	0.978	-0.022	0.949-1.008	0.151
Duration of stimulation	1	0	0.968-1.033	0.995

## Discussion

The present study demonstrated that using a dual trigger for final follicular maturation improved cumulative pregnancy and live-birth rates in each stimulation cycle. Compared with an hCG trigger, a dual trigger also resulted in significantly higher clinical pregnancy, embryo implantation, and live-birth rates in each transfer cycle. Multivariate logistic regression analysis showed that the trigger method was an independent factor affecting the CLBR.

Triggering with GnRHa and hCG (dual trigger) for final follicular maturation during stimulation in IVF/ICSI has recently been suggested to improve treatment outcomes. The dual trigger approach combines the benefits of GnRHa with the long-acting LH activity of hCG. Compared with a single hCG trigger, the addition of GnRHa induces increases in endogenous LH and FSH, similar to the change in hormone levels during the natural ovulation cycle (12). Previous studies confirmed that FSH plays an important role in regulating the formation of LH receptors in granulosa cells (8, 13). Furthermore, the FSH surge could also activate oocyte meiosis, promote the expansion of cumulus granulosa cells, and separate oocytes from the follicle wall, thus facilitating oocyte maturation and ovulation (7, 14). A GnRHa trigger thus improves the rates of oocyte retrieval and maturation during ovarian stimulation. Griffin et al. retrospectively evaluated the oocyte maturation rate in patients with a previous history of retrieval of >25% immature oocytes, and found that the oocyte maturation rate increased significantly after dual trigger in the subsequent stimulation cycle (15). Lu et al. also found that a dual trigger could significantly promote oocyte recovery in low-response patients (9). Haas et al. conducted a double-blind randomised controlled trial comparing the effects of dual and single-hCG triggers on the number of matured oocytes and on pregnancy rate in 150 patients with normal response (1). There were no differences in age, body mass index, and AMH levels between the two groups. Dual trigger significantly increased the numbers of oocytes retrieved, mature metaphase II oocytes, total number of blastocysts, and top-quality blastocysts. Another prospective randomized controlled study also showed that a dual trigger significantly increased the numbers of top-quality embryos and frozen embryos (16). However, in contrast to previous studies, the present study showed that fewer good-quality embryos were obtained in the dual-trigger group compared with the hCG-trigger group. Furthermore, although significantly more oocytes were retrieved in the dual-trigger group, there were no significant differences in oocyte recovery, oocyte maturation, and IVF fertilization rates between the two groups. The difference in the numbers of oocytes retrieved between the groups may be related to the relatively low ovarian reserve in the hCG-trigger group, as well as the higher basal FSH and E2 levels. Meanwhile, Lu et al. found that, although dual trigger significantly promoted oocyte recovery in patients with a suboptimal response, there were no differences in oocyte maturation rates and fertilization rates (9). These inconsistent results among studies may be related to differences in patients and ovarian stimulation cycles.

In addition, a dual-trigger had advantages other than improving oocyte recovery and maturation rates. Lin et al. confirmed that dual trigger significantly improved the embryo implantation and live-birth rates (8), and Schachter et al. demonstrated that dual trigger significantly promoted ongoing pregnancy rates (17). However, these results were all obtained from fresh embryo transfer cycles. The present study thus supplemented the above results by analysing the clinical outcomes of frozen-thawed embryo transfer in freeze-all cycles. The results showed that, although the rate of top-quality embryo transfer in frozen-thawed transfers was lower in the dual-trigger group compared with the hCG-trigger group, the rates of clinical pregnancy, embryo implantation, and live birth were still significantly higher than in the hCG-trigger group. This indicated that the potential competence of embryos obtained in the dual-trigger group was greater than that in the hCG-trigger group. This is an interesting result, given that the rate of good-quality embryos obtained in fresh cycles was lower in the dual-trigger group. This may be because the embryo grade depends on an artificial morphological score assigned by embryologists; this has some limitations, and may not dynamically predict the development potential of the embryos. Furthermore, the better outcomes in terms of pregnancy, implantation, and live-birth rates obtained in frozen-thawed embryo transfer cycles indicated good embryo competence after dual trigger. Consistent with the current results, a previous double-blind randomised controlled study by Haas et al. also showed that dual trigger could provide better ongoing pregnancy, embryo implantation, and live-birth rates (1). In another study, Haas et al. compared the differences in gene expression changes in cumulus granulosa cells after dual and hCG triggers (18). The dual trigger increased the expression levels of both epiregulin and amphiregulin in granulosa cells and decreased the expression of conexin43, indirectly implying that a dual trigger could improve oocyte and embryo competence.

Traditionally, the success of ART has been reported in terms of clinical or ongoing pregnancy rates, implantation rates, and live-births per embryo transfer; however, the CLBR per ovarian stimulation cycle has recently been considered as a better measure of ART success (19, 20). In the present study, we compared the effects of dual and hCG triggers on cumulative pregnancy and live-birth rates, and showed that both were significantly higher after dual trigger. To the best of our knowledge, this study provides the first comparison of cumulative pregnancy and live-birth rates between dual and hCG triggers in freeze-all cycles. We further determined if the effect of dual trigger on the CLBR was influenced by other potential confounding factors using multivariate logistic regression analysis. After controlling for potential confounding variables such as maternal age, number of oocytes retrieved, good-quality embryo rates, fertilization method, and stimulation duration, the trigger method was identified as an independent factor affecting the CLBR, with a dual trigger having a positive effect.

This was a retrospective study including a large sample size, which therefore provided valuable information about the number of oocytes retrieved, number of mature oocytes, and the rates of pregnancy, embryo implantation, live birth, cumulative pregnancy, and cumulative live birth. However, the retrospective study design was associated with inevitable selection bias that might have affected the results. In addition, both ovarian function and the ovarian stimulation protocols differed between the hCG and dual trigger groups in the current study, which may also have affected the results. Nonetheless, dual trigger achieved better clinical outcomes, and this information should be used as a reference indicator during ovarian stimulation in the future.

In conclusion, a dual-trigger approach had a positive effect on CLBRs, suggesting that this should be used as a routine trigger method in freeze-all cycles. To the best of our knowledge, this study provides the first evidence for improved CLBRs using a dual trigger in patients treated with a freeze-all strategy. Further prospective randomized controlled trials are required to verify the beneficial effects of dual trigger on cumulative live births.

## Data Availability Statement

The raw data supporting the conclusions of this article will be made available by the authors, without undue reservation.

## Ethics Statement

The studies involving human participants were reviewed and approved by Reproductive medical Ethics Committee of Sir Run Run Shaw Hospital, College of Medicine, Zhejiang University. The patients/participants provided their written informed consent to participate in this study.

## Author Contributions

HZhu and SZ designed the study. HZhu and CZ drafted the article. YP and HZhou performed statistical analysis. WX and XJ completed data collection. All authors contributed to the article and approved the submitted version.

## Funding

This study was supported by grants from National Key Research and Development Program of China (2018YFC1004800), the Natural Science Program of Zhejiang (LY19H040009), the National Natural Science Foundation of China (No. 81601236).

## Conflict of Interest

The authors declare that the research was conducted in the absence of any commercial or financial relationships that could be construed as a potential conflict of interest.
